# From Safety Climate to Life Satisfaction: The Mediating Role of Job Stress and Job Satisfaction

**DOI:** 10.1016/j.shaw.2025.04.006

**Published:** 2025-04-28

**Authors:** Abdül Halim Özkan, Oğuz Başol

**Affiliations:** 1 Department of Occupational Health and Safety, Kırklareli University, Kırklareli, Türkiye; 2 Department of Business Administration, Bursa Technical University, Bursa, Türkiye

**Keywords:** Job satisfaction, Job stress, Life satisfaction, Occupational health and safety, Safety climate

## Abstract

**Background:**

Healthcare workers and occupational health and safety (OHS) professionals are among the key professions where a strong safety climate is critical. A robust safety climate is theorized to enhance life satisfaction. Both job stress and job satisfaction play influential roles in shaping the effects of safety climate on life satisfaction. This study aims to examine the mediating roles of job stress and job satisfaction in the relationship between safety climate and life satisfaction among healthcare workers and OHS professionals.

**Methods:**

The study utilized data from 610 healthcare workers and OHS professionals employed across Türkiye. Structural equation modelling analysis was performed to explore the relationship between safety climate and life satisfaction and to assess the mediating effects of job stress and job satisfaction.

**Results:**

The findings indicated that job satisfaction serves as a full mediator in the relationship between safety climate and life satisfaction.

**Conclusion:**

Given the mediating role of job satisfaction in the link between safety climate and life satisfaction, organizations should prioritize strategies aimed at improving job satisfaction to enhance overall employee well-being.

## 1 Introduction

In organizational research, the relationship between workplace safety and overall mental health is an area of growing interest. Within this context, the concept of psychosocial safety climate has emerged as a key focus in safety literature. Psychosocial safety climate refers to the policies, practices, and procedures implemented to safeguard employees' psychological health and well-being, highlighting an organization's commitment to its workforce's mental welfare [1]. A well-established safety climate within the workplace not only influences the health and safety of employees [2] but also enhances safety awareness in both professional and personal aspects of their lives [3].

Research indicates that a poor safety climate has detrimental effects on workplace incidents, working conditions for injured employees, and overall employee health [[4], [5], [6]]. This negative environment can adversely impact both job satisfaction and life satisfaction. For instance, studies focusing on healthcare workers have emphasized the critical role of job satisfaction in determining nurses' quality of life [7]. Conversely, low job satisfaction is identified as a leading cause of burnout among healthcare professionals [[8], [9], [10]].

Work occupies a significant place in an individual's life, directly influencing overall life satisfaction. The degree to which a job fulfills psychological, social, and economic needs is closely tied to job satisfaction and, by extension, life satisfaction. Studies show that individuals with high job satisfaction typically experience greater life satisfaction [11]. Conversely, low job satisfaction is often associated with elevated levels of job stress [12]. In a study examining the interplay of job stressors, resources, well-being, and work attitudes among hospital staff, job stress was found to significantly predict perceived stress and job strain, largely by depleting employees' mental and physical resources [13]. High levels of job stress are also strongly linked to symptoms of depression [14] and are among the most critical factors affecting employees' quality of life [15].

Studies on occupational health and safety (OHS) have demonstrated that numerous risk factors affecting healthcare workers also increase the likelihood of errors that can negatively impact patient outcomes. Improving the safety climate for both employees and patients has been shown to enhance healthcare workers’ safety performance while reducing adverse occupational and patient-related outcomes [[16], [17], [18], [19], [20]]. Research on job stress in healthcare settings has further highlighted how conflict management influences stress levels. For example, Johansen and Cadmus found that emergency nurses who avoided conflicts with physicians experienced higher stress levels, while other conflict resolution approaches had no significant effect on stress [21]. Similarly, Tabak and Orit identified that integrative and dominating conflict resolution styles were associated with reduced job stress, whereas coercive and avoidant styles were linked to higher stress levels [22]. Overall, studies suggest that reducing job stress improves employees’ physical and mental well-being, thereby increasing life satisfaction [[23], [24], [25], [26]].

This study aims to explore the impact of safety climate on the life satisfaction of healthcare professionals and occupational safety and health practitioners, including safety specialists, technicians, and physicians. It also investigates the mediating roles of job satisfaction and job stress in this relationship. By focusing on this specific context, the study seeks to address gaps in the literature and provide unique insights into the dynamics between safety climate, job satisfaction, job stress, and life satisfaction.

### 1.1 Research hypothesis

In a study of 6,230 healthcare workers, it was found that reinforcing the safety climate enhances safe behaviors, regardless of psychological or physical safety [27]. Safety climate has also been identified as a factor in unsafe behaviors [[28], [29], [30]]. Research highlights various factors influencing unsafe behaviors, including individual factors such as job stress [[31], [32]], and organizational factors such as safety climate [[33], [34], [35], [36]], safety culture [[37], [38], [39], [40]], and safety management systems [[41], [42], [43]].

Previous studies suggest a positive relationship between health, job satisfaction, and job performance [[44], [45], [46], [47], [48], [49], [50]]. Among nurses, job stress is noted as a psychosocial factor [51] and organizational dynamics often enhances job satisfaction by increasing employee's commitment [52]. The safety climate in the workplace can positively affect life satisfaction, mediated by job stress and job satisfaction.

Hypothesis 1 Safety climate is positively related to job satisfaction.

Safety climate represents employees' shared perceptions of health and safety [44]. In organizations with strong safety climates, management fosters commitment to safety policies, encouraging open communication and feedback. This active participation helps create a healthier workplace [53]. Psychosocial safety climate theory highlights management's commitment to psychological health, prioritization, organizational communication, and participation [54]. Training in psychosocial risks enhances job satisfaction, motivation, and commitment [55]. Strong safety climates ensure both psychosocial and physical well-being, thereby improving job satisfaction [56].

Hypothesis 2 The safety climate is positively related to job stress.

Studies show that safety climate, an organizational factor, correlates with increased injury risks among young employees [57] and mental health challenges among healthcare workers [[58], [59], [60], [61]]. The effort-reward imbalance model highlights that job stress arises when workload exceeds rewards [62,63]. Training and experience in OHS improve awareness and contribute to safety climates, which, in turn, may increase job stress.

Hypothesis 3 Job satisfaction is positively related to life satisfaction.

Research links work-life balance to job satisfaction and mental health [64]. Long working hours disrupt time management and work-life balance [65]. Studies reveal that job satisfaction influences life satisfaction and vice versa [66]. For example, poor job satisfaction reduces job performance in stressful environments [67]. Thus, improving job satisfaction enhances life satisfaction.

Hypothesis 4 Job stress is negatively related to life satisfaction.

Interpersonal conflicts and lack of social support exacerbate job stress [68,69]. Neurological effects of stress can impair emotional regulation, leading to mental health issues like depression [70]. Therefore, job stress diminishes life satisfaction by negatively affecting overall well-being.

Hypothesis 5 Job stress is negatively related to job satisfaction.

The working environment significantly affects mental health [71,72]. Stressful working conditions, such as shift work and long hours, contribute to lower job satisfaction [73]. Among healthcare workers, job stress correlates with reduced satisfaction and increased workplace migration [21,74].

Hypothesis 6 The safety climate is positively related to life satisfaction.

Safety climate influences employee behaviors, with perceptions of safety attitudes mediating satisfaction [75]. A strong safety climate mitigates the negative effects of job risks, improving satisfaction [76]. Studies in mining industries underscore the role of stakeholders, including families, in maintaining a strong safety climate [77]. Improved safety climates contribute to higher life satisfaction.

Hypothesis 7 The mediating role of job satisfaction and job stress in the relationship between safety climate and life satisfaction.

**Fig. 1:**
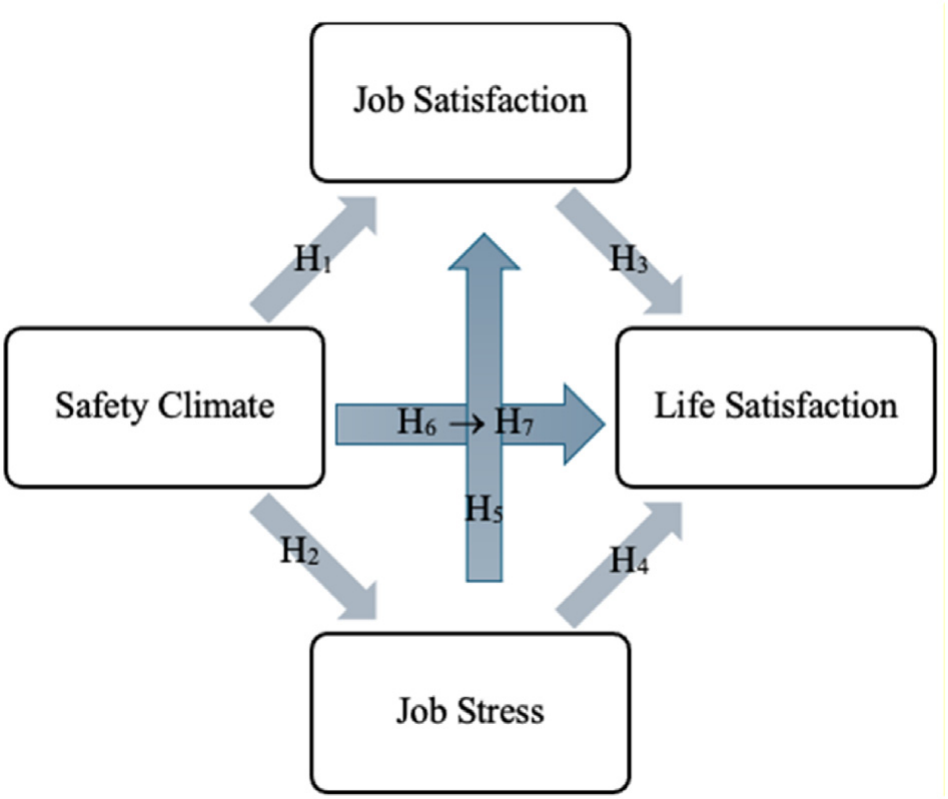
Research model.

Stronger safety climates are linked to higher life satisfaction [48]. Life satisfaction is shaped by both work-related and external factors [78]. Models of job stress [62,79,80] demonstrate its influence on job and life satisfaction. Increased job stress negatively affects life satisfaction by reducing job satisfaction, while enhanced job satisfaction positively impacts life satisfaction. All hypotheses can be seen in Fig. 1.

While existing literature has extensively explored the direct relationships between safety climate, job satisfaction, job stress, and life satisfaction, there remains a significant gap in understanding the underlying mechanisms that connect these variables, particularly within an integrated framework. Prior studies have established that safety climate influences safe behaviors and well-being [27,[33], [34], [35], [36]], and that job satisfaction and job stress individually affect life satisfaction [[44], [45], [46], [47], [48], [49], [50],^62^,^66^]. However, the mediating roles of job satisfaction and job stress in the relationship between safety climate and life satisfaction have not been comprehensively examined together in a single model. Most research focuses on isolated relationships, such as safety climate's effect on job satisfaction [[53], [54], [55], [56]] or job stress's negative impact on well-being [[68], [69], [70]], without fully exploring how these factors interact dynamically to shape employees' overall life satisfaction. This gap is particularly pronounced in high-stakes industries like healthcare, where safety climate is critical, yet its indirect effects on life satisfaction through psychosocial factors remain underexplored.

This study addresses the identified research gap by proposing and testing a comprehensive model that examines the mediating roles of job satisfaction and job stress in the relationship between safety climate and life satisfaction (Hypothesis 7). Our main contribution lies in elucidating how safety climate indirectly influences life satisfaction through two key psychosocial mechanisms: job satisfaction and job stress. By integrating these mediators into a single framework, we provide a nuanced understanding of how a strong safety climate can enhance life satisfaction by fostering higher job satisfaction and mitigating the negative effects of job stress. This dual-mediation approach advances existing theory by demonstrating that safety climate not only directly affects workplace outcomes but also exerts a broader impact on employees' overall well-being through interconnected psychosocial pathways. Drawing on data from healthcare workers–a population highly susceptible to job stress and safety climate influences–this study offers practical insights for organizations aiming to improve employee well-being and life satisfaction by strengthening safety climate initiatives.

## 2 Materials and methods

### 2.1 Participants and procedure

The survey was conducted with the participation of healthcare workers and OHS professionals working in various institutions and organizations across Türkiye. The survey was conducted through online platforms, and 699 people participated in snowball sampling method. The main reason why the snowball sampling method was deemed appropriate for this research is that the research will be conducted in two professional groups and this will facilitate the participants' access to each other. After excluding participants who were not healthcare workers or OHS professionals and 89 questionnaires with incomplete responses, the data of 610 participants were included in the final analysis. Of these, 50.5% of the participants were female (n = 308), 55.2% were married (n = 337), 31.1% had a bachelor's degree (n = 190), and 58.5% were public employees (n = 357). The ages of the participants ranged between 22 and 65 years, with an average age of 36 ± 8.74, and their experience ranged between 2 and 41 years, with an average of 12 ± 8.15 years.

### 2.2 Measures

Safety climate was assessed using the safety climate scale developed by Hahn and Murphy, which consists of 6 items and a single dimension [81]. The Turkish validity and reliability adaptation of the safety climate scale was conducted by Dursun, Başol, and Şengül [82], and Cronbach's Alpha (CA) was calculated as 0.76.

Job satisfaction was assessed using the job satisfaction scale developed by Brayfield and Rothe and shortened by Judge, Locke, Durham, and Kluger, consisting of 5 items and single dimension [83,84]. The Turkish validity and reliability adaptation of the job satisfaction scale was conducted by Başol and Çömlekçi [85] and the CA was calculated as 0.95.

Job stress was assessed using the job stress scale developed by Mulki; Jaramillo, Goad, and Pesquera, which consists of 4 items and a single dimension [86]. The Turkish validity and reliability of the job stress scale was conducted by Dülgeroğlu and Başol [87] and CA was calculated as 0.87.

Life satisfaction was assessed using the life satisfaction scale developed by Diener, Emmons, Larsen, and Griffin, which consists of 5 items and a single dimension [78]. The Turkish validity and reliability of the life satisfaction scale was conducted by Bekmezci and Mert [88], and CA was calculated as 0.90. Lastly, all scales were measured with a 5-point Likert-type rating.

### 2.3 Data analysis

Two different programs were used for data analysis. The data were arranged, and demographic characteristics as well as CA values were calculated as SPSS 21. The structural equation modeling technique was used to test the hypotheses put forward and the analyses were completed with LISREL 8.71.

### 2.4 Ethical considerations

Data collection was carried out after ethics committee permission was obtained. Ethical approval for the study was obtained from the Scientific Research and Publication Ethics Committee of the University (E-35523585-199-141,966). The participants were explained in writing about the purpose of the study before participating in the survey. In addition, only the responses of those who approved of the consent form were accepted. Thus, the questionnaire form prepared for data collection was completed completely by volunteers.

## 3 Result

As a conclusion of our model results, we observed the following: safety climate positively influenced job satisfaction (H_1_) and increased job stress (H_2_). Job satisfaction, in turn, positively impacted life satisfaction (H_3_), whereas job stress had no significant effect on life satisfaction (H_4_). Additionally, job stress negatively influenced job satisfaction (H_5_). We also found that safety climate had a direct positive effect on life satisfaction (H_6_). However, when the mediating roles of job satisfaction and job stress were taken into account, the direct relationship between safety climate and life satisfaction became insignificant (H_7_). This indicates that safety climate does not directly affect life satisfaction but rather influences it indirectly by increasing job satisfaction and job stress. Notably, job satisfaction serves as the key driver of life satisfaction, as job stress does not contribute to life satisfaction.

**Fig. 2:**
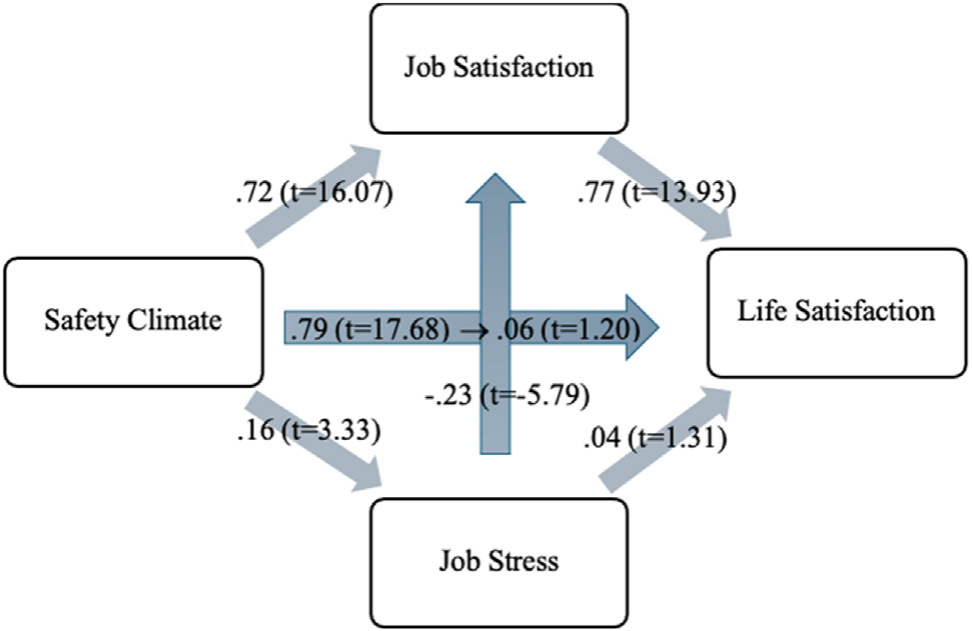
Results of the research model.

In summary, while the creation of a safety climate initially appears to enhance life satisfaction, our findings suggest that this effect is mediated by job satisfaction. Since job stress negatively impacts job satisfaction and does not directly influence life satisfaction, job satisfaction emerges as the critical determinant of life satisfaction. Given that the goodness-of-fit statistics for the proposed model are appropriate, we conclude that the model is statistically significant. The results of the current research model are shown in Fig. 2.

In order for the obtained model results to be considered significant, the model should also be examined in terms of goodness-of-fit statistic values. When the estimated was analyzed, we found that the values were within acceptable limits: (Χ^2^/df = 4.74 < 5), p value (p < .01), root mean square error of approximation (RMSEA) (<.08), standardized root mean square residual (SRMR) (<.08), normed fit index (NFI) (>.95), non-normed fit index (NNFI) (>.95), comparative fit index (CFI) (>.95), incremental fit index (IFI) (>.95), relative fit index (RFI) (>.95), goodness of fit index (GFI) (>.90), and adjusted goodness of fit index (AGFI) (>.85) (Table 1).

**Table 1:** Goodness-of-fit statistics of the model

Chi-square	df	*p*	RMSEA	SRMR	NFI	NNFI	CFI	IFI	RFI	GFI	AGFI
678.16	143	0.00	0.078	0.069	0.96	0.97	0.97	0.97	0.96	0.90	0.86

Table 2 shows the model results and the status of the hypotheses. According to the results, all hypotheses were found to be significant except H_4_.

**Table 2:** Model results

Path	β	t	Hypothesis
Safety climate → job satisfaction	0.72	16.07	H_1_
Safety climate → job stress	0.16	3.33	H_2_
Job satisfaction → life satisfaction	0.77	13.93	H_3_
Job stress → life satisfaction	0.04	1.31	H_4_
Job stress → job satisfaction	-.23	-5.79	H_5_
Safety climate → life satisfaction	0.79	17.68	H_6_
Safety climate → job satisfaction, job stress→ life satisfaction	0.06	1.20	H_7_

## 4 Discussion

This research examines the mediating roles of job satisfaction and job stress in the relationship between safety climate and life satisfaction. The findings indicate a full mediation effect. Specifically, while job satisfaction mediates the relationship between safety climate and life satisfaction, job satisfaction is negatively impacted by job stress and positively influenced by safety climate. The findings showed that the strengthening of the safety climate for employees increased both job stress and job satisfaction. This is in line with the results in the literature for job satisfaction [[4], [5], [6]]. Similar to previous studies, the findings related to job stress show that it reduces employee’s job satisfaction [12,73,74,76]. However, while studies in the literature show that job stress decreases life satisfaction [14,15,[23], [24], [25], [26],^79^] and both life satisfaction and job satisfaction [71,73,87,88], our findings revealed that there was no significant relationship between them. The finding of a positive relationship between job satisfaction and life satisfaction is in line with the studies in literature. When the mediating role of job stress and job satisfaction in the relationship between safety climate and life satisfaction is taken into account, it is revealed that there is no direct relationship between safety climate and life satisfaction.

In light of the findings, it is suggested that organizations aiming to increase employee welfare should first develop strategies to improve job satisfaction levels. For this purpose, it is important to revise remuneration policies, offer career development opportunities, and strengthen communication both among employees and between managers and employees. In addition, organizations should take proactive steps to reduce work-related stress by clarifying roles and responsibilities, creating transparent work policies and regulating working hours. Moreover, it is imperative that OHS is adopted as one of the organization's key policy priorities and that high standards are maintained in both implementation processes and related regulations. Considering that a positive safety climate in the workplace directly affects employees' life satisfaction and job satisfaction plays a crucial role in this interaction, strengthening employees' intrinsic and extrinsic motivational resources will further support this relationship. This situation increases the importance of motivational practices to be implemented at the organizational level in working life. By managing job stress levels and ensuring job satisfaction, the potential effects of the safety climate, which was initially created by taking costs into consideration, can be made more apparent.

The research was conducted with healthcare and OHS professionals in Türkiye, and the analyses are based solely on specific scales and the scanning electron microscopy technique. This means that the findings may vary in other sectors, in different geographical regions, or when alternative methodological approaches are adopted. Future research should take these contextual and methodological differences into account in order to confirm the validity and broaden the scope of these results.

While this study focused on healthcare and OHS professionals, future research could also examine other specialized groups such as specialists, academics, teachers, engineers, etc. Furthermore, cross-country comparisons with nations at similar levels of development may provide a broader perspective. Extending the model to include moderating variables, such as managerial roles or previous experience with occupational accidents, could provide a better understanding of these relationships.

### 4.1 Limitations of the study

As with all surveys, this study was conducted based on the personal declaration of the participants. The only limitation of the study is that the participants included in the sample are occupational physicians, occupational health personnel, occupational safety specialists, or healthcare workers working in the field of OHS. Healthcare workers and OHS professionals were included in the study together since they are the two occupational groups in which the understanding of safety climate in Türkiye is most effective.

## CRediT authorship contribution statement

**Abdül Halim Özkan:** Conceptualization; Data curation; Investigation; Methodology; Software; Supervision; Validation; Visualization; Writing - review and editing. **Oğuz Başol:** Formal analysis; Methodology.

## Conflicts of interest

The authors declared no conflicts of interest.
